# Fusion Entrapment Enables Unbiased Assessment of False Discovery Rate Control in Cascaded Database Searches

**DOI:** 10.1016/j.mcpro.2026.101636

**Published:** 2026-08-11

**Authors:** Xinpei Yi, Yan Fu

**Affiliations:** 1 National Facility for Protein Science in Shanghai, Shanghai Advanced Research Institute, Chinese Academy of Sciences, Shanghai, China; 2 University of Chinese Academy of Sciences, Beijing, China; 3 State Key Laboratory of Mathematical Science, Academy of Mathematics and Systems Science, Chinese Academy of Sciences, Beijing, China

**Keywords:** Cascaded database search, false discovery rate, fusion entrapment, target-decoy apporach, metaproteomics

## Abstract

Cascaded database searches boost identification sensitivity in vast proteomic search spaces but challenge false discovery rate (FDR) control. The standard target-decoy approach to FDR control relies on decoy matches providing an exchangeable and properly scaled representation of incorrect target matches. This assumption can be disrupted when protein-level filtering is used to define a reduced search space, because target and decoy entries may no longer undergo symmetric retention during database reduction. Although entrapment provides an external benchmark for assessing FDR control, conventional separate-entrapment implementations can become invalid in cascaded searches because entrapment sequences may be disproportionately discarded during protein-level filtering. Here we introduce Fusion Entrapment, a strategy that computationally fuses entrapment sequences with target proteins to preserve identical selection pressure during database reduction. Simulations show that this strategy provides accurate entrapment-based false discovery proportion (FDP) estimation in cascaded searches involving protein-level filtering. Applying Fusion Entrapment to human gut metaproteomic datasets, we further observed that conventional separate target-decoy database reduction led to substantial inflation of the entrapment-estimated FDP relative to the reported FDR threshold. In contrast, fusion target-decoy reduction maintained empirical FDR control under Fusion Entrapment assessment while retaining substantial sensitivity gains over single-step analysis.

## Highlights

• Identifying biased entrapment depletion in cascaded database searches.
• Revealing separate-entrapment failure with ground-truth simulation.
• Fusing entrapment entries to preserve symmetric protein-level filtering.
• Improving metaproteomic sensitivity while maintaining FDR control.

## Graphical Abstract

**Figure:**
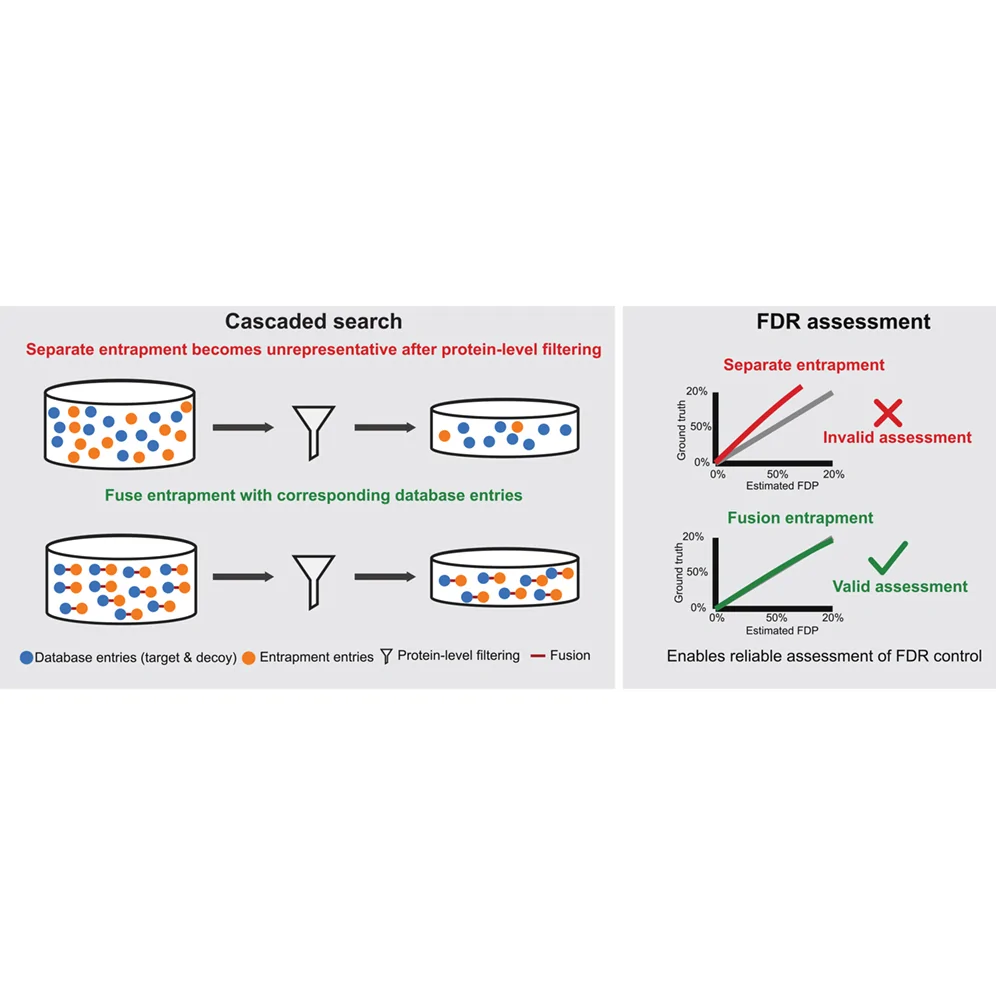

## In Brief

We introduce Fusion Entrapment, an entrapment-based strategy for assessing FDR control in cascaded database searches involving protein-level database reduction. By fusing entrapment sequences with corresponding targets, Fusion Entrapment preserves symmetric selection during database filtering and prevents biased depletion of entrapment entries. Simulations with known ground truth and analysis of human gut metaproteomic datasets show that Fusion Entrapment can accurately assess FDR control while retaining the sensitivity gains of cascaded searches.

In shotgun proteomics, the identification of peptides and proteins relies heavily on matching tandem mass spectrometry (MS/MS) spectra against sequence databases. A pervasive challenge in this field, particularly in metaproteomics, is the vast explosion of the search space. As databases scale up, the increased probability of random matches creates a high noise background that obscures true signals. Rigorous false discovery rate (FDR) control inevitably leads to a substantial loss in identification sensitivity (1). To resolve this trade-off between search space size and sensitivity, cascaded (or iterative) search strategies have become a popular solution (2, 3, 4, 5). These strategies typically adopt an initial screening against the full database to identify plausible candidate proteins, which are then used to construct a reduced database. A subsequent search against this refined search space significantly boosts identification sensitivity (6).

While highly effective for maximizing identifications, cascaded strategies introduce complex statistical challenges regarding FDR control (7). The target-decoy approach (TDA) remains the standard strategy for controlling FDR in peptide identification (8, 9), although decoy-free methods have also been proposed (10). The effectiveness of TDA relies on the assumption that decoy matches provide an appropriate proxy for incorrect target matches, including comparable score behavior and proportional representation of false matches. However, this assumption is not universally satisfied. For instance, in the identification of peptide subgroups, subgroup-specific error proportions or score behavior may differ from the global mixture, so a global FDR estimate can misrepresent subgroup-specific error rates (11, 12, 13). Importantly, as critically highlighted by Bern and Kil (14), cascaded search procedures can also violate this assumption. Specifically, the initial search acts as a filter that preferentially retains target proteins exhibiting sufficient identification evidence (e.g., multiple high-scoring peptide matches). Protein-level filtering after the initial search conditions the second-step target database on first-step identification evidence. Target entries are preferentially retained when they are supported by true sample-derived peptide evidence, while a smaller subset of absent target entries may also pass the filter by chance through stochastic high-scoring peptide-spectrum matches (PSMs). In contrast, independently handled decoy entries are not guaranteed to undergo the same conditional retention process: carried-over decoys must themselves accumulate sufficient random evidence to survive protein-level filtering, whereas regenerated decoys are created only after target-side database reduction. Consequently, the decoy search space entering the second-step search can be disproportionately depleted or differently conditioned relative to the retained target search space. When spectra are searched again against this imbalanced reduced database, incorrect PSMs are more likely to be assigned to target entries, leading to underestimated FDR (7, 14). This asymmetric depletion violates the fundamental TDA assumption that decoy matches provide a properly scaled proxy for false target matches in the analyzed search space, potentially leading to an accumulation of false positive identifications that go unnoticed (15, 16).

To evaluate the validity of FDR control, the entrapment strategy has been widely adopted (17, 18). This strategy introduces sequences known to be absent from the sample (e.g., from foreign species) into the database to guarantee that any identification matching these sequences can be unambiguously classified as a false positive, thus serving as a ground-truth proxy for error monitoring. However, despite its widespread use, its implementation has been inconsistent and often methodologically flawed. Previous studies frequently relied on flawed estimators that merely provided a lower bound for the true error rate. Because such lower bounds capture only a fraction of all false positives, they can reveal severe FDR inflation when they exceed the reported threshold, but they cannot establish valid FDR control when they fall below it. This limitation has led to a lack of consensus on the actual effectiveness of FDR control in proteomics. Recently, Wen *et al*. (19) established a formal statistical framework for entrapment analysis, revealing that many state-of-the-art proteomics tools fail to accurately control FDR, and proposing rigorous estimators (e.g., the paired and combined methods) to estimate error rates. However, applying the entrapment strategy to cascaded searches poses a fundamental challenge rooted in database construction. As conceptually illustrated in Supplementary Fig. S1, the score distribution of matches to entrapment sequences initially approximates that of false target PSMs before database reduction. However, this statistical correspondence can be disrupted under conventional separate filtering at the protein level. Because entrapment sequences are physically absent from the sample, matches to them arise only from stochastic incorrect PSMs. After the initial search, protein-level screening is performed based on the first-step search results to construct a reduced database for the second-step search. Under separate entrapment, target and entrapment entries are screened independently. Target entries can satisfy the screening criterion either because they are supported by true sample-derived peptide evidence or through stochastic incorrect matches. In contrast, entrapment entries can be retained only if they themselves accumulate sufficient random evidence. As a result, entrapment entries are disproportionately discarded relative to target entries during database reduction, leaving a severely depleted entrapment search space for the second-step search. In the second-step search, spectra are searched again against this reduced database, and the false target PSMs ultimately observed in the final results may include both erroneous matches reproduced from the initial search and additional, potentially more numerous, erroneous matches generated during the second-step search. Because these false target PSMs occur within the retained target-side search space, whereas the independently retained entrapment search space has been disproportionately depleted, separate entrapment can no longer provide a representative benchmark for the false target PSMs in the final results, leading to underestimation of the final FDP. Thus, for cascaded search workflows that use protein-level filtering or protein-based database reduction, a critical methodological gap remains in the rigorous assessment of FDR control. Although the present study focuses on cascaded searches with protein-level database reduction, similar selection-induced biases may also arise in other workflows that incorporate protein-level selection, filters, or features, including machine learning-based rescoring pipelines (20). These considerations highlight the need for proper entrapment designs that preserve symmetric selection between target and entrapment entries, particularly in workflows where protein-level information influences the peptide identification process.

Fusion-based database designs have been used previously to restore statistical symmetry in several proteomics contexts. Zhang *et al*. (21) introduced the decoy fusion strategy, in which target and decoy sequences are concatenated within the same database entry, to reduce bias in target-decoy-based FDR estimation when protein-level information influences peptide identification. Ivanov *et al*. (22) later adapted this idea for existing multi-stage search workflows, showing that fused decoy database designs can mitigate biased target-decoy selection during secondary searches. More recently, Solivais *et al*. (23) used fused entrapment sequences to assess peptide-identification errors in peptide-identity propagation, and Bogdanow *et al*. (24) applied target-decoy fusion to cross-linking mass spectrometry to improve error estimation under context-sensitive filtering. These studies establish that structural coupling of target, decoy, or entrapment entries can be valuable when downstream filtering or scoring steps introduce asymmetric selection.

The present study focuses on the distinct but related problem of entrapment-based assessment of FDR control in cascaded searches involving protein-level database reduction. In this setting, conventional separate entrapment entries are filtered independently and can be preferentially removed before the second-step search. As a result, the retained entrapment search space no longer provides a representative benchmark for the false target PSMs ultimately observed in the final second-step results. To our knowledge, this failure mode has not been systematically characterized for cascaded searches, nor has fusion-based entrapment been benchmarked as an FDR-assessment protocol for this setting. We therefore adapt the fusion principle to entrapment-based FDR assessment by coupling entrapment sequences with their corresponding target entries during database reduction. Using the paired method introduced by Wen *et al*. (19) as the primary FDP estimator, we assess the Fusion Entrapment strategy through simulations with known ground truth and through reanalysis of complex human gut metaproteomic datasets. Together, this Fusion Entrapment workflow enables systematic assessment of whether cascaded search strategies involving protein-level filtering maintain valid FDR control while retaining their sensitivity advantage.

## Experimental Procedures

### Construction of Simulated Datasets

To rigorously evaluate the performance of FDR control strategies, we generated synthetic tandem mass spectrometry (MS/MS) datasets with known ground truth. We generated 100 replicate datasets to ensure statistical robustness, with each replicate created independently as described below. We started with a pool of 408,340 synthetic protein sequences designed to mimic the statistical properties (e.g., amino acid distribution and length) of the human proteome (11). For each simulation replicate, proteins were randomly selected from this pool and assigned to three distinct categories to model different identification scenarios:

True Target Proteins (Candidates): 2000 proteins were randomly selected to represent “true” proteins present in the sample. Their sequences were included in the search database, and peptides derived from these proteins were used as inputs for in silico MS/MS spectral prediction. The predicted spectra were subsequently subjected to noise injection to obtain simulated MS/MS spectra.

Noise Proteins: 700 proteins were randomly selected to represent “noise” or contaminants that might generate sectra, but whose sequences are not present in the search database. This simulates the presence of unidentifiable spectra or “dark matter” in proteomics data.

Others: 100,000 proteins were randomly selected to serve as background entries in the search database. While their sequences were included in the database, no spectra were generated from them. These proteins challenge the search engine's ability to distinguish true matches from random database hits.

Proteins in the “True Target” and “Noise” categories were subjected to in silico digestion using trypsin specificity. We allowed for up to two missed cleavages and filtered the resulting peptides to retain only those with lengths between 7 and 35 amino acids. From the resulting peptide pools, we applied a weighted random sampling strategy to select peptides for spectral prediction. This procedure was performed separately for the “True Target” and “Noise” protein pools. For each protein, duplicate peptide sequences were first removed, and the number of available unique peptides was recorded. Let $n_{i}$ denote the number of available unique peptides from protein *i*. We defined an unnormalized protein-level allocation weight as.

$$a_{i}=\sqrt{n_{i}}$$

and converted it into a normalized sampling proportion,

$$p_{i}=\frac{a_{i}}{\sum_{j}a_{j}}$$

Thus, proteins with more available peptides were assigned larger sampling quotas, but the square-root transformation reduced the dominance of long or peptide-rich proteins. For a target of *N* sampled peptides in a given pool, the expected quota for protein *i* was calculated as $Np_{i}$. Integer protein-level peptide quotas were then assigned using a largest-remainder apportionment procedure and were capped by the number of available peptides for each protein. Peptides were sampled without replacement within each protein, while enforcing global uniqueness of peptide sequences across proteins. If duplicate peptide sequences across proteins reduced the total number of selected peptides, the remaining quota was filled by randomly sampling from the remaining globally unique peptides. Using this procedure, we selected 20,000 unique peptides from the 2000 “True Target” proteins and 10,000 unique peptides from the 700 “Noise” proteins for subsequent spectral prediction.

Theoretical MS/MS spectra for the selected peptides were predicted using AlphaPeptDeep (25) with default parameters. To mimic the complexity and imperfections of real experimental data, we developed a custom Python script (mgf_noise_injector.py) to inject realistic noise and artifacts into the predicted spectra. The noise injection process included the following perturbations:

Fragment Loss: Random removal of theoretical fragment peaks with a loss rate of 25%, while protecting the top 10 most intense peaks to preserve spectral identity.

Signal Jitter: Introduction of random fluctuations to both mass-to-charge ratios (m/z) and intensities. m/z values were jittered by 8 ppm, and intensities were varied by 25%.

Random Noise Peaks: Addition of random noise peaks to each spectrum, consisting of 50 low-intensity peaks and 8 high-intensity peaks.

Chimeric Spectra: To simulate co-fragmentation events, we introduced chimeric spectra with a probability of 0.5. Donor peaks were selected from other spectra within a 1.5 Da precursor m/z window, and the top 10 peaks from the donor were added with an intensity scaled to 20% of the original.

Precursor Mass Errors: We simulated precursor mass measurement errors using a mixed Gaussian and Laplace distribution and introduced isotope selection errors with a probability of 8%.

Peak Merging: Nearby peaks within a tolerance of 0.02 Da were merged to simulate resolution limits.

This procedure resulted in a final set of ∼30,000 spectra (∼20,000 from targets and ∼10,000 from noise) for each replicate. The corresponding source protein database for each replicate contained the 2000 “True Target” protein sequences and the 100,000 “Other” protein sequences, totaling 102,000 protein entries.

### Metaproteomic Datasets

To evaluate the performance of the Fusion Entrapment strategy on real-world complex samples, two public human gut metaproteomic datasets were reanalyzed. The first dataset was derived from PXD003528, which originally comprised eight human mucosal-luminal interface (MLI) samples collected from pediatric patients (26). The mass spectrometry data were acquired on a Q Exactive mass spectrometer (Thermo Fisher Scientific) using a 4-h LC gradient. In the original study, this dataset yielded 67,186 distinct peptide sequences corresponding to 19,011 protein groups, with a median identification rate of 32% and a median of 15,210 identified peptide sequences per sample (26). In the present re-analysis, three raw files were selected from this dataset (Supplementary Table S1). The second dataset was derived from PXD008738, which originally included six human fecal samples from healthy individuals (27). These samples were analyzed on a Q Exactive HF mass spectrometer (Thermo Fisher Scientific) using a 90-min LC gradient. Because the present study evaluates DDA-style peptide-spectrum matching and database search-based FDR control, we used the six DDA library raw files generated from pooled human fecal samples (Supplementary Table S1). The original study reported 12,804 unique peptides from the human fecal samples, with unique taxonomic annotations assigned to 47% of peptides at the phylum level and 28% at the genus level (27). In the present re-analysis, both datasets were searched against the human gut microbial Integrated Gene Catalog (IGC; 9,878,647 sequences), representing a large and heterogeneous microbial search space. The combination of complex human gut microbial samples, deep peptide/protein identification in the original studies, and the multi-million sequence search database supports the use of these datasets as representative complex metaproteomic benchmarks for evaluating FDR-control strategies in cascaded searches.

### Comet Database Search

We employed Comet (version 2025.02) to perform database searching for both simulated and real-world datasets, adapting the search parameters to the specific nature of each dataset. For the simulated datasets, the 2000 true target proteins and 100,000 background proteins were used as the source protein database for defining ground truth and protein-level filtering. For Comet searching, this source protein database was converted into an in silico digested peptide-level FASTA database, while retaining the mapping between each peptide entry and its originating protein. The simulation search was therefore configured without enzyme specificity (no digestion) and with no missed cleavages. No fixed or variable modifications were applied. After the first-step search, peptide-spectrum matches were mapped back to their source proteins, and protein-level database reduction was performed using the retained protein identifiers. Thus, although the simulated Comet search was performed against peptide-level FASTA entries, the cascaded reduction step was implemented at the protein level. For the real-world metaproteomic datasets (PXD003528 and PXD008738), the raw files were searched against the human gut microbial Integrated Gene Catalog (IGC; 9,878,647 sequences). The entrapment and decoy databases were generated using the strategy described in the Entrapment Database Generation section. The search parameters were configured as follows: enzyme specificity was set to trypsin with a maximum of two missed cleavages; precursor mass tolerance was 10 ppm; and fragment mass tolerance was 0.02 Da. Carbamidomethylation of cysteine was set as a fixed modification, and oxidation of methionine was allowed as a variable modification with a maximum of five modifications per peptide. For all analyses, the primary cross-correlation score (XCorr) reported by Comet was used for downstream target-decoy competition and entrapment-based FDR assessment.

### Entrapment Database Generation

To rigorously evaluate FDR control, we generated entrapment sequences using the FDRBench software (19). For both simulated and real-world datasets, we employed a peptide-level entrapment strategy. Specifically, for each original target peptide sequence in the database (T), a corresponding entrapment peptide (E) was generated by randomly shuffling the amino acid sequence while preserving the C-terminal amino acid (to maintain tryptic digestion properties) and ensuring the new sequence did not exist in the original database. Subsequently, decoy sequences were generated for both the original targets (D_T_) and the entrapment targets (D_E_) using the reverse sequence method. This process resulted in a composite database containing four components: Original Target (T), Entrapment Target (E), Original Decoy (D_T_), and Entrapment Decoy (D_E_). For the Separate Entrapment implementation, the four components were treated as independent database entries during protein-level filtering. For the Fusion Entrapment implementation, each entrapment entry was computationally linked to its corresponding original target entry, and each entrapment-decoy entry was linked to its corresponding original-decoy entry, forming paired T-E and D_T_-D_E_ units for database reduction. This linkage was used only to determine retention during database reduction. In the second-step search and final FDRBench assessment, peptide labels were mapped back to their original T, E, D_T_, or D_E_ categories and kept distinct.

### FDR Control Evaluation Procedures

We evaluated the FDR control performance of cascaded search strategies by integrating the search engine results with the FDRBench evaluation module. The workflow consisted of the following steps:

1. One-Step Search Baseline.

As a quality control baseline, the mass spectra were searched against the composite database (T + E + D_T_ + D_E_) in a single pass. The search results were input into FDRBench to calculate the False Discovery Proportion (FDP) using the paired method. This step verified the intrinsic quality of the dataset and the validity of the entrapment generation under the same primary estimator used in the downstream analyses.

2. Two-Step Search Strategies.

To mimic the cascaded workflows used in metaproteomics, we performed a two-step search involving a coarse filtering step followed by a refined search. We implemented and compared two distinct Target-Decoy (TD) filtering strategies during the first step:

Separate TD Strategy: In this conventional approach, protein-level filtering was performed independently for the target and decoy subsets. In this study, a protein entry was retained in the reduced database only if it was supported by at least two unique peptides based strictly on evidence from its own entry type. Consequently, an entrapment protein could be discarded even if its paired original target protein was retained, and vice versa.

Fusion TD Strategy: Fusion TD refers to the target-decoy filtering strategy during database reduction, rather than to the construction of the entrapment database. In this strategy, each target-side entry was coupled with its corresponding decoy-side entry during protein-level filtering. Under Fusion Entrapment, the target-side entry was the fused target-entrapment pair (T-E), and the decoy-side entry was the fused decoy pair (D_T_-D_E_). Filtering evidence, such as unique peptide counts, was pooled across the target-side and decoy-side counterparts. If the paired unit satisfied the filtering criterion, both counterparts were retained in the reduced database; otherwise, both were discarded. This symmetric retention rule prevents target and decoy entries from undergoing different selection pressures during cascaded database reduction. This pooling was used only to determine whether paired target-side and decoy-side entries were retained during the database-reduction step. In the second-step search and final FDR assessment, target, entrapment, and decoy labels were kept distinct, and peptide-spectrum matches were scored and evaluated according to their original labels.

3. FDR Evaluation.

Following the construction of the reduced databases using either the Separate or Fusion strategy, a second-step search was performed against each reduced database. The final search results were parsed and analyzed using FDRBench. We used the paired method as the primary FDP estimator because it provides a tighter upper-bound estimate for entrapment-based FDR assessment. The estimated FDP was then compared with the reported FDR threshold (e.g., 1%) to classify the outcome as “Test passed” when the estimated FDP did not exceed the reported FDR threshold, or as “Test failed” when the estimated FDP exceeded the reported FDR threshold.

## Results

### The Fusion Entrapment Strategy for Rigorous FDR Assessment in Cascaded Searches

**Fig. 1:**
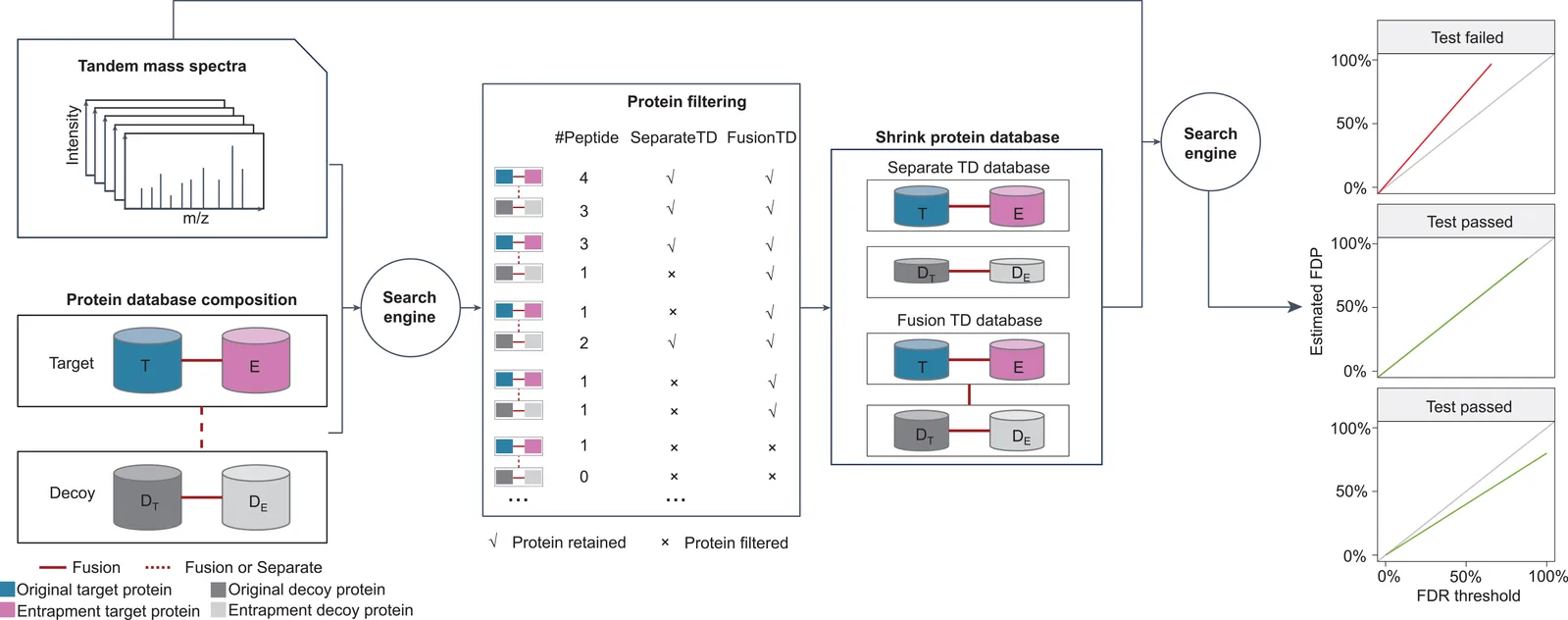
**Schematic workflow of the Fusion Entrapment strategy for assessing FDR control in cascaded database searches.** The workflow begins with protein database construction, in which original target proteins (T) are computationally coupled with entrapment target proteins (E) so that both components experience the same selection pressure during protein-level filtering. Corresponding decoy entries are generated for the original target and entrapment sequences (D_T_ and D_E_). Tandem mass spectra are first searched against the initial comprehensive database, and the resulting identifications are then used for protein-level filtering to construct a reduced database for the second-step search. The central table illustrates the difference between the Separate TD and Fusion TD filtering strategies. In Separate TD, target-side and decoy-side entries are filtered independently, which can lead to asymmetric retention of paired entries. In Fusion TD, the filtering evidence for each target-side entry and its corresponding decoy-side entry is pooled, so that both counterparts are retained or discarded together. After database reduction, a second-step search is performed against the reduced database. The final search results are evaluated using FDRBench with the paired method as the primary FDP estimator. The *right*-hand schematic illustrates the paired-estimator-based decision rule used in this study: if the estimated FDP apparently exceeds the reported FDR threshold, the strategy is classified as “Test failed”; otherwise, the strategy is classified as “Test passed.” The *gray* diagonal line represents y = x, where the estimated FDP equals the reported FDR threshold.

To address the methodological challenge of assessing FDR control in cascaded database searches, we developed the Fusion Entrapment strategy (Fig. 1). As illustrated in the workflow, the process begins with database construction. First, rather than treating entrapment sequences as independent entries, we employ a fusion strategy where entrapment target sequences (E) are computationally fused with original target protein sequences (T). This structural linkage ensures that, during database reduction, an entrapment component paired with an original target protein is retained or discarded together with that target, rather than being filtered as an independent entry. Second, a decoy database is constructed based on this fused target set (T + E).

Our analysis involves evaluating two distinct Target-Decoy (TD) implementations during the filtering stage: Separate TD and Fusion TD. Following the initial search by the search engine, protein-level filtering is performed to generate a reduced database. Figure 1 details this filtering logic using a representative threshold of 2 unique peptides per protein. In the Separate TD approach, targets (T + E) and decoys (D_T_ + D_E_) are filtered independently. For instance, if a target has 1 identified peptide and its corresponding decoy has 1 identified peptide, both fail to meet the threshold of 2 (marked as ×), leading to the removal of both from the reduced database. In contrast, the Fusion TD approach couples the target and decoy. In the same scenario (1 target peptide, 1 decoy peptide), the combined count is 2. Since this meets the threshold, both the target and decoy are retained (marked as √). Based on these filtering results, a reduced protein database is constructed for each strategy. A second-step search is then performed against these reduced databases. The final search results are then evaluated using FDRBench (19), with the paired method used as the primary FDP estimator. The estimated FDP is compared with the reported FDR threshold according to the decision rule shown in Figure 1: if the estimated FDP exceeds the reported FDR threshold, the strategy is classified as “Test failed”; otherwise, the strategy is classified as “Test passed”.

### Assessment of Fusion Entrapment Using Large-Scale Simulations

**Fig. 2:**
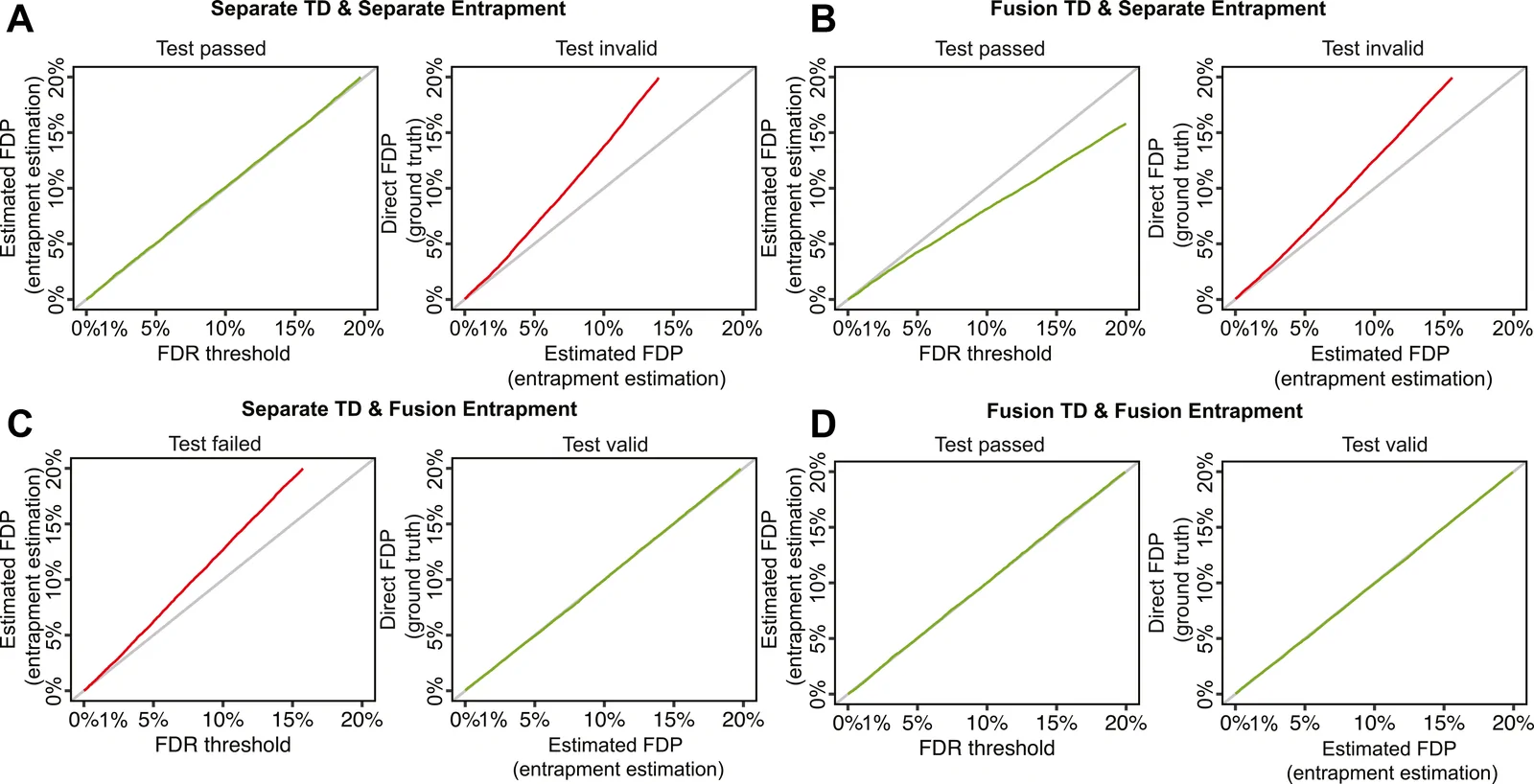
**Assessment of the Fusion Entrapment strategy across 100 simulation replicates using synthetic datasets with known ground truth. Each panel displays two plots.** The *left* plot compares the FDP estimated by the paired method against the reported FDR threshold, assessing FDR control according to the decision rule shown in Figure 1. The *right* plot compares the direct FDP calculated from ground truth against the FDP estimated by entrapment, assessing whether the entrapment-based estimate accurately reflects the true error rate. Colored curves represent the mean FDP values across 100 simulation replicates. *Green* curves indicate “Test passed” or “Test valid” results, whereas *red* curves indicate “Test failed” or “Test invalid” results. The *gray* diagonal line represents y = x. *A*, results for the Separate TD search strategy assessed using Separate Entrapment. The *left* plot suggests apparent FDR control, but the *right* plot shows that the entrapment-estimated FDP deviates from the direct FDP, indicating that Separate Entrapment does not accurately estimate the true error *rate* and is therefore invalid in this cascaded setting. *B*, results for the Fusion TD search strategy assessed using Separate Entrapment. Although the left plot again suggests apparent FDR control, the *right* plot shows that the entrapment-estimated FDP does not match the direct FDP, indicating that Separate Entrapment remains invalid after cascaded database reduction. *C*, results for the Separate TD search strategy assessed using Fusion Entrapment. The *right* plot shows close agreement between the entrapment-estimated FDP and the direct FDP, supporting the validity of Fusion Entrapment. In the *left* plot, the estimated FDP exceeds the reported FDR threshold, supporting a “Test failed” classification and indicating that Separate TD does not adequately control FDR in this simulation setting. D. Results for the Fusion TD search strategy assessed using Fusion Entrapment. The *right* plot shows close agreement between the entrapment-estimated FDP and the direct FDP, supporting accurate entrapment-based FDR assessment. In the *left* plot, the estimated FDP closely follows the reported FDR threshold, supporting a “Test passed” classification and indicating accurate FDR control by the Fusion TD strategy.

We first assessed the performance of our strategy in a controlled simulation environment with known ground truth. To ensure statistical robustness, we performed 100 simulation replicates (Experimental Procedures). As a baseline quality control, we first evaluated the performance in a standard One-Step search. As shown in Supplementary Fig. S2, both the entrapment-estimated FDP and the direct FDP calculated from ground truth were closely aligned with the theoretical FDR threshold (y = x diagonal), supporting the reliability of our simulation dataset and the validity of the entrapment methodology in a standard one-step setting. Next, we evaluated the performance in a Two-Step search scenario (Fig. 2). We compared four combinations of strategies: Separate TD and Separate Entrapment (Fig. 2*A*), Fusion TD and Separate Entrapment (Fig. 2*B*), Separate TD and Fusion Entrapment (Fig. 2*C*), and Fusion TD and Fusion Entrapment (Fig. 2*D*).

To simplify interpretation and to follow the primary estimator emphasized in the FDRBench framework (19), the simulation results were visualized using the paired method. Wen *et al*. (19) showed that the paired method provides a tighter upper-bound FDP estimate, and we therefore used it as the primary estimator for both FDR-control assessment and evaluation of entrapment-estimation validity. In the right-hand assessment plots, the agreement between the estimated FDP and the direct FDP calculated from ground truth was used to assess whether the entrapment estimate itself was valid.

The results reveal a critical distinction. When Separate Entrapment was used (Fig. 2, *A* and *B*), the left plots suggested apparent FDR control, but the right plots showed that the entrapment-estimated FDP deviated from the direct FDP, indicating that Separate Entrapment failed to accurately represent the true error rate in the cascaded-search setting. In contrast, Fusion Entrapment produced FDP estimates that closely matched the direct FDP, confirming the validity of Fusion Entrapment. When assessed by Fusion Entrapment, the Separate TD strategy showed entrapment-estimated FDP values exceeding the reported FDR threshold (Fig. 2*C*), supporting a “Test failed” classification. Conversely, the Fusion TD strategy showed close alignment with the diagonal and was classified as “Test passed” (Fig. 2*D*), indicating that the search strategy maintained FDR control and that Fusion Entrapment provided an accurate FDR assessment.

Collectively, these simulations show that, within the cascaded-search setting involving protein-level filtering examined here, Fusion Entrapment yielded entrapment-based error estimates that agreed with the ground-truth error rate, whereas separate entrapment failed to accurately represent the true error rate after protein-level database reduction.

### Application to Real-World Metaproteomic Datasets

**Fig. 3:**
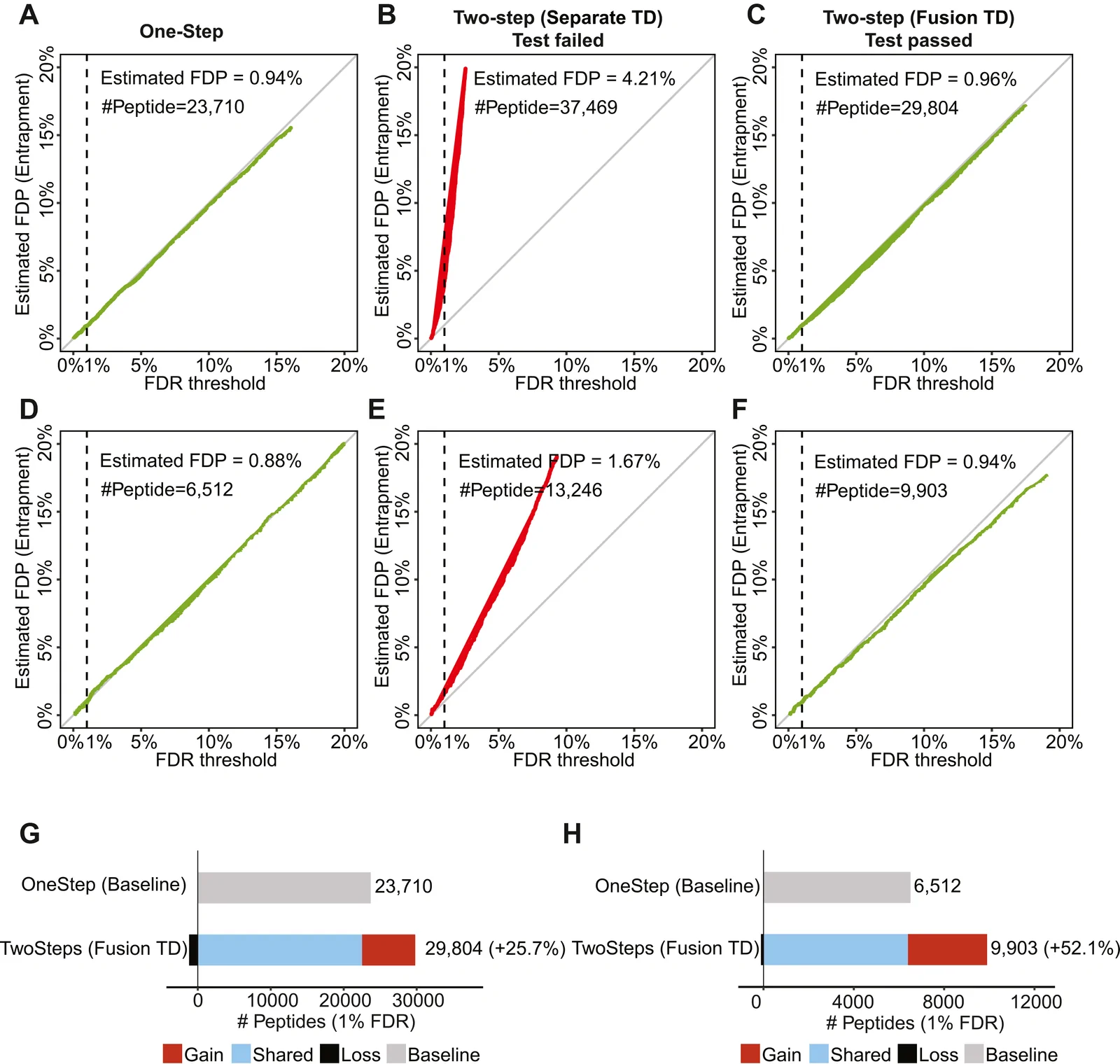
**Application of Fusion Entrapment to assess FDR control and sensitivity in real-world metaproteomic datasets.** Two public human gut metaproteomic datasets were analyzed to benchmark cascaded search strategies. *Panels A–C* correspond to Dataset 1, and *panels D–F* correspond to Dataset 2. In *panels A–F*, the curves show the entrapment-estimated FDP, calculated using the paired method as the primary estimator for FDR-control assessment. *Green* curves indicate results classified as valid FDR control or “Test passed,” whereas *red* curves indicate results classified as “Test failed.” The gray diagonal line represents y = x, and the dashed vertical line indicates the 1% FDR threshold. Text insets display the estimated FDP at the 1% FDR threshold and the total number of identified unique peptides. *A, D*, baseline One-Step search results. The estimated FDP closely follows the reported FDR threshold, supporting valid FDR control in the One-Step search setting. *B, E*, two-step search using the Separate TD filtering strategy. The estimated FDP exceeds the reported FDR threshold, supporting a “Test failed” classification when assessed by Fusion Entrapment and suggesting FDR inflation under Separate TD filtering. *C, F*, two-step search using the Fusion TD filtering strategy. The estimated FDP closely tracks the reported FDR threshold, supporting a “Test passed” classification when assessed by Fusion Entrapment and suggesting valid FDR control. *G, H*, comparison of identification sensitivity at 1% FDR between the One-Step baseline and the valid Two-Step Fusion TD strategy for Dataset 1 (*G*) and Dataset 2 (*H*). The stacked bars illustrate the overlap between methods: peptides shared with the baseline, new peptides gained by the Two-Step strategy, and peptides lost. The total number of peptides and the percentage increase relative to the baseline are indicated.

Having assessed the performance of the Fusion Entrapment strategy in simulations with known ground truth, we next applied it to real-world large-scale metaproteomic datasets for FDR-control assessment. We analyzed two distinct public datasets (Experimental Procedures) to evaluate the performance of cascaded searches. First, we examined the standard One-Step search to ensure baseline consistency. As shown in Figure 3, *A* and *D*, the entrapment-estimated FDP aligns well with the reported target-decoy FDR threshold, confirming the applicability of our assessment approach to complex samples.

Next, we used Fusion Entrapment to evaluate Two-Step searches using either Separate TD or Fusion TD strategies for database construction. Consistent with our simulation results, the Separate TD strategy showed substantial inflation of the entrapment-estimated FDP relative to the reported FDR threshold in both datasets. As evidenced by Figure 3, *B* and *E* (“Two-step: Test failed”), the entrapment-estimated FDP curves exceeded the reported FDR threshold, suggesting that independent filtering of targets and decoys can lead to underestimated error rates in cascaded searches involving protein-level database reduction. In contrast, the Fusion TD strategy produced estimated FDP curves that closely tracked the diagonal (Fig. 3, *C* and *F*, Test passed), supporting empirical FDR control when assessed by Fusion Entrapment. Furthermore, at the 1% FDR threshold, the Fusion TD strategy yielded a substantial increase in peptide identifications compared to the One-Step baseline (+25.7% for Dataset 1 and +52.1% for Dataset 2, Fig. 3, *G* and *H*), demonstrating the practical value of cascaded searches when database reduction is performed symmetrically.

In conclusion, consistent with previous reports that two-step or cascaded database searches can improve identification sensitivity compared to one-step searches in large search spaces (2, 5, 6), the metaproteomic analyses here show that these sensitivity gains depend critically on how the reduced database is constructed. In the datasets examined, Fusion TD retained the identification advantage of cascaded searching while supporting valid FDR control when assessed by Fusion Entrapment, whereas Separate TD filtering showed evidence of FDR inflation.

## Discussion

Cascaded database searches are widely used to improve sensitivity in large proteomic search spaces, particularly in metaproteomics. Expanding the candidate space increases the number of random high-scoring matches, forcing stricter error control and thereby penalizing sensitivity. To address this, cascaded searches iteratively reduce the candidate pool, enabling a final search on a smaller, higher-confidence subset and improving identifications. However, database reduction can compromise the validity of FDR control. More precisely, the TDA assumption that the number of decoy matches mirrors that of incorrect target matches can be violated when cascaded workflows perform protein-level database reduction using multi-round searches without enforcing symmetric treatment of target and decoy entries.

While the entrapment strategy serves as a benchmark for assessing FDR reliability, its implementation presents unique methodological challenges. Recent work by Wen *et al*. (19) formalized entrapment analysis for single-step searches, providing statistically valid upper and lower bounds on the FDP. However, we show that these entrapment estimators can become invalid in cascaded-search workflows. The problem is not the estimators themselves but the database construction. During the initial filtering, entrapment sequences are disproportionately removed, making them unrepresentative of false target PSMs in the final refinement step. As a result, standard entrapment protocols may fail to detect selection-induced bias in cascaded workflows, limiting their utility for reliable FDR control assessment.

To address this, we introduced Fusion Entrapment, which fuses entrapment entries to their paired target proteins during database reduction. By enforcing identical protein-level retention criteria on each target–entrapment pair, Fusion Entrapment preserves the representativeness of entrapment entries for assessing FDR control in cascaded searches. Across extensive simulations, Fusion Entrapment reliably detected error inflation in cascaded workflows, whereas separate entrapment implementations failed to identify this bias.

Applying this strategy to real-world metaproteomic datasets showed that independent filtering of targets and decoys during cascaded database reduction can lead to underestimated error rates and potential false-positive inflation. Conversely, the fusion-based strategy, which couples targets and decoys during database reduction, supported empirical FDR control when assessed by Fusion Entrapment while retaining the sensitivity advantage of cascaded searching. These results suggest that independent filtering in cascaded workflows may have led to underestimated error rates in prior studies.

Taken together, the novelty of Fusion Entrapment does not lie in the general concept of sequence fusion itself, which has been used previously in target-decoy FDR estimation and related error-assessment settings, but rather in its application and systematic evaluation as an entrapment-based strategy for assessing FDR control in cascaded searches involving protein-level database reduction. By comparing separate and fusion-based implementations under both simulated ground truth and real metaproteomic datasets, this study defines the specific failure mode of conventional entrapment for FDR assessment in cascaded workflows and provides a practical protocol for preserving entrapment validity under protein-level filtering. The current evidence therefore most directly supports the use of Fusion Entrapment in cascaded workflows where protein-level filtering or protein-based database reduction defines the second-step search space. Similar selection-induced biases may also arise in other workflows where protein-level information influences filtering (28), scoring (20), or search-space reduction (29), and Fusion Entrapment should be applicable to these workflows too, but such settings were not directly examined here and require future workflow-specific evaluation.

## Code Availability

The source code for reproducing the results and figures is available under an Apache license via GitHub at https://github.com/YiCITI/FusionEntrapment.

## Data Availability

The simulated MS/MS datasets and source data used to reproduce the figures are available via Zenodo at https://zenodo.org/records/18640430. The public metaproteomic raw datasets re-analyzed in this study are available through the ProteomeXchange Consortium/PRIDE under accession numbers PXD003528 and PXD008738. All datasets are publicly accessible and do not require login credentials.

## Supplemental data

This article contains supplemental data.

## Conflict of interest

The authors declare that they have no conflicts of interest with the contents of this article.
