# Supplementary material for: Fusion Entrapment Enables Unbiased Assessment of False Discovery Rate Control in Cascaded Database Searches
Source: Mol Cell Proteomics. 2026 Aug 11;25(9):101636. doi: 10.1016/j.mcpro.2026.101636 (PMC13563576; doi:10.1016/j.mcpro.2026.101636)
Supplement: Supplementary file 1 [file mmc1.pdf]

Supplemental Figures

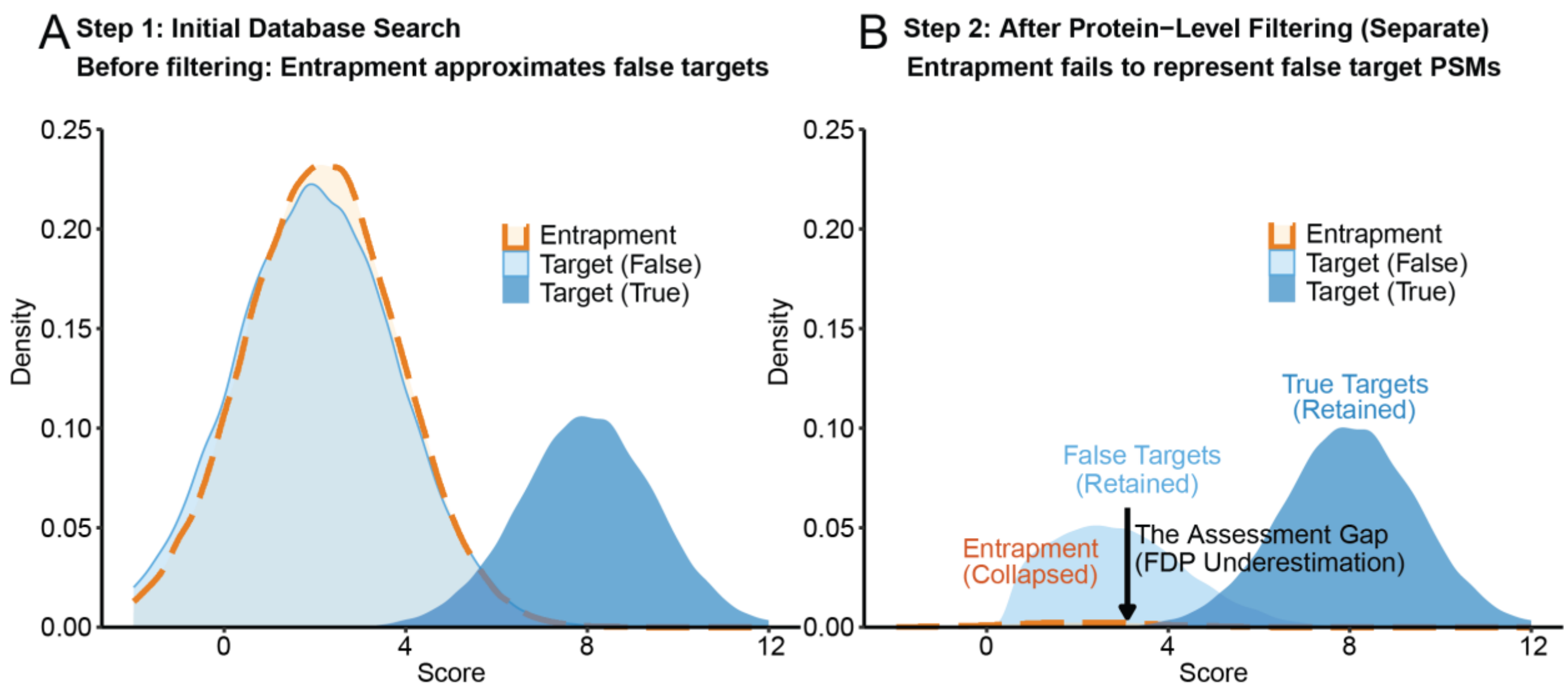

Supplementary Figure 1. Conceptual mechanism of entrapment-based FDR assessment failure under separate filtering. A. Step 1: Initial database search score distributions. The score distribution of matches to entrapment sequences broadly overlaps with that of false target PSMs, indicating that entrapment initially serves as an approximate statistical proxy for false target PSMs before database reduction. True target matches form a distinct high-score population. In panel A, the entrapment distribution is shown as a light orange density with a dashed orange outline to indicate its overlap with the false-target distribution, whereas the false-target and true-target distributions are shown as filled density curves. B. Step 2: Asymmetric depletion after protein-level filtering. Under the conventional separate filtering strategy, protein-level screening is performed using evidence from the initial search to construct the reduced database for the second-step search. Target entries can be retained either because they are supported by true sample-derived peptide evidence or, less frequently, because absent target entries accumulate sufficient stochastic incorrect PSMs. In contrast, entrapment entries are absent from the sample and, when filtered independently, can be retained only through stochastic incorrect PSMs. As a result, entrapment

entries are disproportionately discarded relative to target entries during database reduction, leaving a severely depleted entrapment search space in the second-step database. During the second-step search, spectra are searched again against this reduced database, and the final false target PSMs may include both erroneous matches that were also observed in the initial search and additional, potentially more numerous, erroneous matches generated during the second-step search. Because these errors occur within the retained target-side search space, whereas the independently retained entrapment search space has been depleted, separate entrapment no longer provides a representative benchmark for the final false target PSMs. This asymmetric depletion creates an assessment gap, indicated by the black arrow, leading to underestimation of the final FDP. The y-axis represents score density, and the x-axis represents the search engine score.

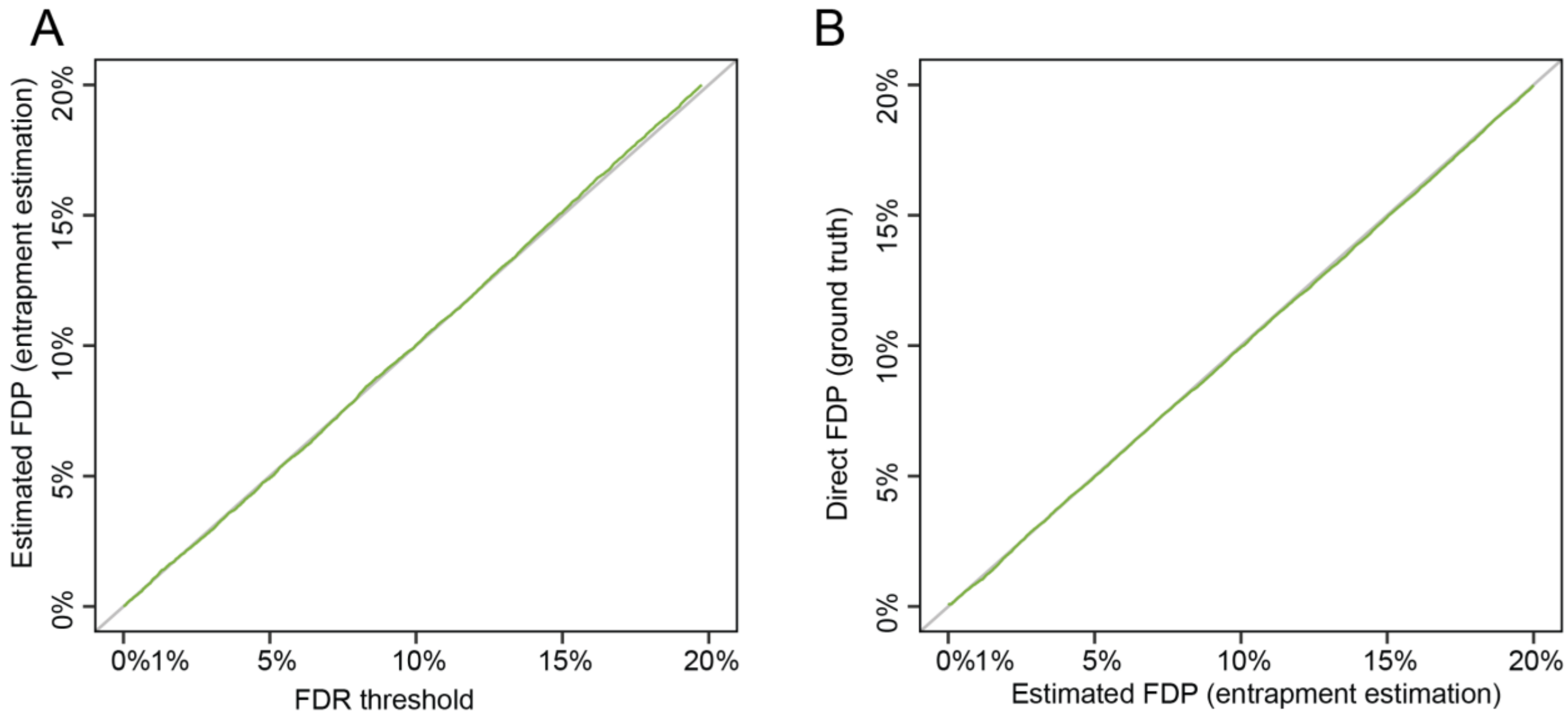

Supplementary Figure 2. Baseline assessment of entrapment-based FDP estimation in a standard one-step database-search scenario using 100 simulation replicates with known ground truth. A. The entrapment-estimated FDP, calculated using the paired method, is compared with the reported FDR threshold. The close alignment of the green curve with the gray diagonal line indicates that the one-step search controls the FDR appropriately in this baseline setting. B. The direct FDP calculated from ground truth is compared with the FDP estimated by entrapment, assessing whether the entrapment-based estimate accurately reflects the true error rate. In both panels, green curves represent the mean FDP values across 100 simulation replicates. The gray diagonal line represents y = x.
